# ASO Author Reflections: Impact of Preoperative MRI on Patients With Screen-Detected Invasive Breast Cancer Undergoing Breast-Conserving Surgery

**DOI:** 10.1245/s10434-021-09936-6

**Published:** 2021-04-11

**Authors:** Jessie J. J. Gommers, Lucien E. M. Duijm, Adri C. Voogd

**Affiliations:** 1 Department of Medical Imaging, Radboud University Medical Center, Nijmegen, The Netherlands; 2 Department of Radiology, Canisius Wilhelmina Hospital, Nijmegen, The Netherlands; 3 Department of Epidemiology, Maastricht University, Maastricht, The Netherlands

## Past

Breast-conserving surgery (BCS) is a standard surgical treatment for early-stage breast cancer. The primary goal of BCS is to obtain complete tumor excision because positive resection margins are found to be associated with an increased risk of local recurrence.^1^ If tumor excision is incomplete, re-excision or mastectomy may be necessary, which can result in increased physical and emotional burden for the patient as well as extra health care costs. Preoperative assessment to determine the extent of breast cancer is crucial for complete tumor excision. Breast magnetic resonance imaging (MRI) is widely used for the preoperative evaluation of tumor extent, and findings have shown this method to be better than mammography or ultrasonography for evaluating tumor size and delineating tumor margins.^2^ The high sensitivity of breast MRI for cancer detection is presumed to improve surgical planning and decrease the rates of a positive resection margin at the first BCS attempt.^3^ However, most studies investigating the impact of preoperative MRI on surgical margin status have included patients with clinically detected breast cancer, and data on the value of preoperative breast MRI in screen-detected breast cancer are lacking. Cancers detected during screening usually are non-palpable and smaller than clinically detected breast cancers. The exact localization of these screen-detected cancers is thus very important to the achievement of complete tumor excision.

## Present

Using multivariable analysis, this study found that selective use of preoperative MRI was associated with a lower risk of positive resection margins (involved margins > 4 mm) after BCS for patients with screen-detected invasive breast cancer (adjusted odds ratio [OR], 0.56; 95% confidence interval [CI], 0.33–0.96).^4^ Moreover, the presence of microcalcifications (adjusted OR, 4.45; 95% CI, 2.69–7.37), architectural distortions (adjusted OR, 1.85; 95% CI, 1.01–3.40), high (> 75 %) mammographic breast density (adjusted OR, 3.61; 95% CI, 1.07–12.12), lobular histology (adjusted OR, 2.86; 95% CI, 1.68–4.87), and increasing tumor size (per mm of increase; adjusted OR, 1.05; 95% CI, 1.03–1.07) were independently associated with positive resection margins after BCS. Because most of these factors can be assessed preoperatively, they may improve surgical planning and thereby margin status after BCS. Consequently, it may be argued that preoperative MRI should be performed in high-risk settings involving microcalcifications, architectural distortions, high (>75 %) breast density, large tumors, and lobular histology.

## Future

The study results suggest that selective use of preoperative MRI for women with screen-detected invasive breast cancer promises to minimize the risk of positive resection margins after BCS. Future studies should focus on the prognostic impact of the study findings and investigate the cost-effectiveness of preoperative breast MRI for surgical planning.
